# The ADH1B Arg47His polymorphism in East Asian populations and expansion of rice domestication in history

**DOI:** 10.1186/1471-2148-10-15

**Published:** 2010-01-20

**Authors:** Yi Peng, Hong Shi, Xue-bin Qi, Chun-jie Xiao, Hua Zhong, Run-lin Z Ma, Bing Su

**Affiliations:** 1 State Key Laboratory of Genetic Resources and Evolution, Kunming Institute of Zoology and Kunming Primate Research Centre, Chinese Academy of Sciences, Kunming, China; 2 Human Genetics Centre, School of Life Science, Yunnan University, Kunming, China; 3 Institute of Genetics and Developmental Biology, Chinese Academy of Sciences, Beijing, China; 4 Graduate School of Chinese Academy Sciences, Beijing, China

## Abstract

**Background:**

The emergence of agriculture about 10,000 years ago marks a dramatic change in human evolutionary history. The diet shift in agriculture societies might have a great impact on the genetic makeup of Neolithic human populations. The regionally restricted enrichment of the class I alcohol dehydrogenase sequence polymorphism (ADH1BArg47His) in southern China and the adjacent areas suggests Darwinian positive selection on this genetic locus during Neolithic time though the driving force is yet to be disclosed.

**Results:**

We studied a total of 38 populations (2,275 individuals) including Han Chinese, Tibetan and other ethnic populations across China. The geographic distribution of the ADH1B*47His allele in these populations indicates a clear east-to-west cline, and it is dominant in south-eastern populations but rare in Tibetan populations. The molecular dating suggests that the emergence of the ADH1B*47His allele occurred about 10,000~7,000 years ago.

**Conclusion:**

We present genetic evidence of selection on the ADH1BArg47His polymorphism caused by the emergence and expansion of rice domestication in East Asia. The geographic distribution of the ADH1B*47His allele in East Asia is consistent with the unearthed culture relic sites of rice domestication in China. The estimated origin time of ADH1B*47His allele in those populations coincides with the time of origin and expansion of Neolithic agriculture in southern China.

## Background

The major diet shift in recent human history was caused by domestication of plants and animals[1]. During human evolution, diet shifts may create different selective pressures acting on the genetic variations of human populations. Two well-studied examples are the copy number variation of amylase gene for starchy food and the regulatory sequence variations of lactase for milk [2-4]. In southern China, the earliest agriculture started to flourish due to the domestication of rice about 10,000 years ago [5]. Hence, like the amylase gene selected for high copy numbers in agricultural societies including East Asia, the rice-culture-related selection could have been acting on populations living in southern China. Rice has been used as the material to produce fermented food and beverages for a long time in southern China since early Neolithic time. The fermentation helps to preserve and enhance the nutritional value of foods and beverages[6]. However, alcohol can lead to addiction and cause damages to human bodies, including nervous system dysfunction, tumor genesis, innate immune system modulation and fetal alcohol syndrome [7-11]. Therefore, genes involved in the ethanol metabolic pathway might become the target of selection when the ethanol-containing food and beverages had been routinely consumed by Neolithic populations in southern China.

The Class I alcohol dehydrogenase (ADH) is the major enzyme that catalyzes alcohol to acetaldehyde in liver. The Class I ADH genes (ADH1A, ADH1B, and ADH1C) encode three subunits of Class I ADH isoenzymes, i.e. α, β and γ. The well studied sequence polymorphism, ADH1BArg47His (rs1229984) is located in ADH1B. The change of amino acid from Arg to His causes enzymatic activity alteration. The derived allele, ADH1B*47His, changes the pKa of the enzyme from 8.5 to 10.0 which is associated with 40 to 100 fold increase in K_m _and V_max _of alcohol metabolism [12,13]. A global investigation of the ADH1B*47His allele frequency shows a strong geographic distribution. It is dominant in East Asian populations, but rare in European and African populations[14]. The molecular signature of positive selection on ADH1B have been reported [15,16], and the culture-related selective forces were proposed [17] though no correlation with rice domestication has been tested. We hypothesize that the emergence and expansion of rice domestication during Neolithic time is the driving force, leading to the current regional distribution of the ADH1BArg47His polymorphism in East Asia.

## Results

### ADH1B*47His allele frequency in East Asian populations

**Figure 1:**
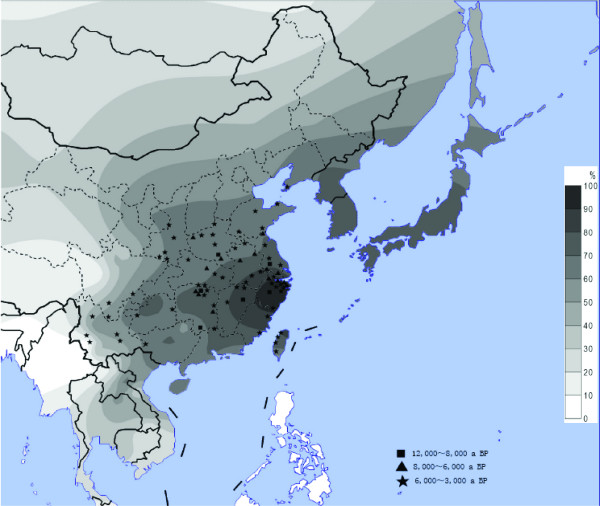
The distribution of the ADH1B*47His allele and the sites of early rice relics. The contour map of the ADH1B*47His frequency in East Asian populations and the ancient sites of rice domestication in China. The allele frequency data includes the 38 populations in the present study and those published before.[17]. The geographic locations of the rice sites are from the published data [5].

We analyzed a total of 2,275 individuals from 38 East Asian populations, especially those not included in the previous reports (northern Han Chinese, Tibetan and southern ethnic populations in China). Table 1 lists the frequencies of ADH1B*47His in the 38 populations. In general, the distribution pattern is consistent with the previous reports[14,17], and most of the populations (31/38) have frequencies higher than 50%. In Han Chinese, the highest frequency is detected in Zhejiang province of south-eastern China (98.5%), and those in the west have relatively low frequencies (60-70%). The same pattern is also observed for the other ethnic populations from China and Southeast Asia (Cambodia and Thailand) except for Tibetan (14.1% on average), Bulang (1.7%, an ethnic population from south-western China) and Cambodian (20.6%). All the five Tibetan populations from different geographic regions have low frequencies (13-21%). We created a contour map based on the data from the 38 populations and those published before (Figure. 1). The distribution of the frequencies of ADH1B*47His confirms its prevalence in East Asia and a clear east-to-west cline is observed.

**Table 1:** The distribution of the ADH1B*47His allele in the 38 East Asian populations.

Population	Long. (E)	Lat. (N)	Sample Size	Language	ADH1B*47His Frequency (%)
**Han Chinese**			679		66.7
Jilin	125	44	14	Han	50
Gansu	105	36	174	Han	59.8
Shanxi	112	37.5	44	Han	61.4
Shannxi	108	34.5	52	Han	62.5
Shandong	120	37	90	Han	63.3
Henan	112	34	56	Han	64.3
Anhui	118	33	34	Han	66.2
Liaonin	124	42	28	Han	67.9
Hubei	113	30	41	Han	70.7
Jiangsu	119	34	24	Han	70.8
Guangxi	109	24	9	Han	72.2
Guizhou	107	28	61	Han	75.4
Hunan	110	27.5	20	Han	82.5
Zhejiang	121	30	32	Han	98.5
**Tibetan**			1088		14.1
Shannan	92	29	34	Tibeto-Burman	11.8
Dangxiong	91	30.5	816	Tibeto-Burman	13.1
Changdou	97	31	56	Tibeto-Burman	14.3
Rikaze	89	29	55	Tibeto-Burman	16.4
Qinghai	96	33	127	Tibeto-Burman	20.5
**Bulang**	100	23.5	30	Austro-Asiatic	1.7
**Cambodian**	105	13	17	Austro-Asiatic	20.6
**Hani**	102.5	23	30	Tibeto-Burman	41.7
**Bai**	100	25.5	30	Tibeto-Burman	50
**Yi**	101.5	24.5	30	Tibeto-Burman	55
**Tujia**	109	30	31	Tibeto-Burman	66.1
**Dai**	100	23.5	30	Daic	43.3
**Maonan**	108	25	15	Daic	60
**Buyi**	106.5	26	45	Daic	63.3
**Shui**	108	26	11	Daic	66.7
**Chuang**	108	23	33	Daic	72.7
**Gelo**	106	27	10	Daic	75
**Mulam**	109	24	12	Daic	75
**Dong**			58		65.5
Hunan	110	27.5	42	Daic	64.3
Guangxi	109	26	16	Daic	68.7
**Yao**	111	25	58	Hmong-Mien	64.7
**Miao**	109	26	29	Hmong-Mien	70.7
**She**	118	27	12	Hmong-Mien	91.7
**Manchu**	125	42.5	27	Altai	66.7

### Selection on the ADH1B gene

**Figure 2:**
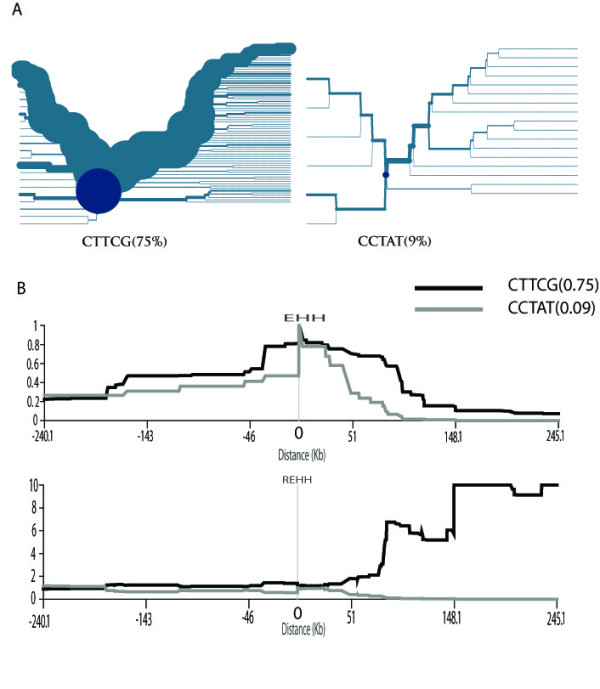
**The selection test for the ADH1B gene region**. (A) The haplotype-bifurcation diagrams for the core haplotype with at least 9% frequency at the ADH1B gene region in the East Asian populations. The core haplotype CTTCG shows unusual long-range homozygosity. (B) The EHH and REHH plots of the core haplotype covering the candidate SNP (rs1229984) in the East Asian populations. The EHH and REHH values are plotted against the physical distances extending both upstream and downstream of the selected core region. Only the core haplotypes with frequency ≥9% are shown.

To detect the molecular signature of recent selection on the ADH1B*47 polymorphism, we applied the LRH method and the iHS statistics using the genotype data from the HapMap project. The obtained iHS value for the core SNP (rs1229984) is -2.189 (the empirical p-value is 0.0269), an indication of selection. We then define the core region of ADH1B on the basis of five SNPs (rs4147536, rs1229984, rs1353621, rs1159918 and rs6810842) which determine the East Asian-dominant haplotype. We also select the flanking SNPs, extending both upstream and downstream to 250 Kb, to study the decay of LD from the core haplotype. We plot the haplotype-bifurcation diagrams[18] for the two East Asian populations (Figure. 2) from HapMap (JPT: Japanese in Tokyo, Japan; CHB: Han Chinese in Beijing, China). At a minimum threshold of 9%, we define two core-region haplotypes in the JPT+CHB population. The haplotype CTTCG, which covers the derived variant of ADH1B*47His has an extended predominance by showing a thick branch in the haplotype-bifurcation diagram, clearly suggesting a long-range LD.

The EHH and REHH of the major core haplotypes (≥9%) are plotted against the distance away from the core for the JPT+CHB population (Figure. 2). The EHH of the CTTCG core haplotype decays more slowly than that of the other core haplotype (containing the ancestral variant ADH1B*47Arg) does. In addition, the upstream REHH value of the CTTCG is 17.329 (P = 0.01, by using 1-NORMSDIST). Again, this result is highly consistent with the previous studies, in which the molecular signature of selection was suggested in a wider genomic region containing the ADH1B locus among East Asian populations[15,17]. The selection on ADH1B was also reported previously when the global populations were screened[16]. Additionally, a strong signature of positive selection was detected for the ADH gene cluster in a genome-wide analysis[19]. Collectively, the distribution of ADH1B*47His allele frequency in the populations studied cannot be explained by random genetic drift, and recent selection needs to be invoked.

### The time of selection

**Figure 3:**
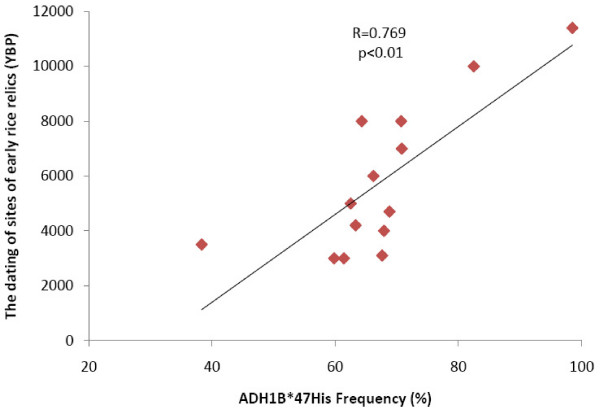
The correlation test for ADH1B*47His allele frequencies with the ages of rice domestication. The correlation of the ADH1B*47His allele frequencies with the ages of rice domestication in 14 regions of China. The data of rice domestication was collected from the published study [5]. The correlation analysis was conducted with the use of SPSS13.0, and the statistical significance was accessed by t test.

Previous studies suggested a culture-related selection on the ADH1B*47His[17]. To test this, we superimposed the unearthed culture relic sites of rice domestication in East Asia and we observed a significant correlation of the ADH1B*47His allele frequencies with the ages of rice domestication (r = 0.769, p < 0.01, two-tailed t test; Figure 3; see Additional file 1). The origin of rice domestication occurred along the Yangtze River of southern China about 10,000 years ago[20,21]. Based on the culture relics, the earliest rice sites are located in southern and south-eastern China (8,000-12,000 YBP), and then expanded to the central parts of China about 3,000-6,000 years ago, reaching Korea and Japan less than 3,000 years ago [22,23]. The spread of rice domestication agrees well with the distribution of ADH1B*47His, implying that rice domestication is likely the force driving up the frequency and expansion of ADH1B*47His in East Asia during the past 10,000 years. To see if the initial increase of ADH1B*47His in East Asia occurred during the same period as the emergence of rice domestication in early Neolithic time, we conducted molecular dating[24] by typing the nearest STR loci (a CATA repeat STR located about 14 Kb upstream to the ADH1B locus, and a ATTC repeat STR located about 35 Kb downstream to the ADH1B locus) in 598 individuals randomly selected from the 38 populations. For phase reconstruction, only homozygous individuals with the ADH1B*47His alleles are included (see Additional file 2). The estimated ages based on the STRs are 5,525 YBP (CATA repeat), and 9,200 YBP (ATTC repeat). Considering that the two STR loci are still far away from the ADH1B locus, we also estimate the age of the ADH1B*47His based on the phased SNP haplotypes from the HapMap dataset (see Additional file 3). With the fine-scale genetic map, we selected 19 contiguous polymorphic SNPs to estimate the age (Table 2). Surprisingly, the estimated ages are extremely different between the upstream SNPs (114,693-208,919 yrs, 95% confidence interval) and the downstream SNPs (7,338-9,948 yrs, 95% confidence interval), which is due to the dramatic change of recombination rates in the studied genomic region. As suggested, the method based on the moments estimator[24] is not suitable for the region of low average recombination rates. The previous genomic study based on the HapMap SNPs also excluded the regions with low average recombination rate[25]. Therefore, the age estimated based on the downstream SNPs seems to reflect the real age of ADH1B*47His allele, which is also consistent with the ages estimated from the STR variations. Taken together, the age of the derived allele at the ADH1B locus falls in the range of 10,000-7,000 years before present.

**Table 2:** Estimated allele ages for ADH1B*47His.

			*SNP Frequency(%)*		
				
*Polymorphism*	Distance to ADH1B*47His (bp)	Genetic distance to ADH1B*47His (cM)	G	A	*Allele Age(years)*
**Upstream SNP**					
rs4147531	-27122	0.011537	80.95	89.86	164,811
rs1229966	-25886	0.009885	66.67	89.86	91,782
rs12507573	-12995	0.007675	80.95	89.86	247,742
rs1042026	-10853	0.007588	66.67	89.86	119,567
rs17033	-10374	0.007568	80.95	89.86	251,245
rs2066701	-906	0.007073	66.67	89.86	128,272
rs2075633	-321	0.007021	66.67	89.86	129,222
**Average**					**161,806 (SD = 63,596)**
**Downstream SNP**					
rs1159918	3690	0.007325	33.33	98.55	7,494
rs9307239	7618	0.053403	47.62	86.96	13,406
rs2213041	8032	0.054139	90.48	98.55	7,621
rs1789891	11100	0.055169	90.48	98.55	7,479
rs1789982	11339	0.055228	90.48	98.55	7,470
rs2173201	11651	0.055302	38.10	85.51	12,059
rs1154433	14389	0.056568	90.48	98.55	7,294
rs1229978	16880	0.056640	90.48	98.55	7,284
rs2298753	18588	0.056670	90.48	98.55	7,281
rs1614972	18838	0.056678	38.10	85.51	11,765
rs1789898	19017	0.056679	90.48	98.55	7,280
rs1662060	20522	0.056693	90.48	98.55	7,279
**Average**					**8,643 (SD = 2,307)**

## Discussion

Having established that the rice culture is likely the driving force of selection on the ADH1BArg47His polymorphism, the left question would be to explain the selective advantage of the ADH1B*47His allele. In southern China, people began to make fermented beverages long time ago. The potential benefits of having fermented beverage (or foods) can be explained by ethanol's combined analgesic, disinfectant and profound mind-altering effects[26]. In addition, fermentation helps to preserve and enhance the nutritional value of foods and beverages. Chemical analyses of ancient organics absorbed into pottery jars suggests that the earliest production of rice fermentation was carried out by the Neolithic people who lived in southern China about 9,000 years ago[6], not long after the origin of rice domestication in the same region. We believe that the custom could have prevailed rapidly among those early-agriculture populations in southern China during the Neolithic time, which have lasted thousands of years.

The ADH I has a low K_m _for ethanol, found in the liver, which metabolizes the most part of ethanol in the body. The derived ADH1B*47His allele is known to metabolize ethanol up to 100 times quicker than the ancestral ADH1B*47Arg allele, providing support that quick eradication of ethanol, and therefore lower local exposure should be protective. The recent case-control studies also suggested that the ADH1B*47His allele is the protective variant [27-30]. The higher metabolic rate of ADH1B*47His may also lead to the accumulation of the toxic aldehyde intermediate that has been commonly associated with the flushing phenotype[31]. An association study in Han Chinese indicates that the individuals carrying ADH1B*47His have the lowest risk for alcoholism[32]. It was suggested that the flushing phenotype is biochemically equivalent to the effects of disulfiram (a drug used to prevent relapse)[33], which can influence drinking behaviour as a way of protection from over consumption of alcohol. It can also protect against the damage to human bodies caused by alcohol consumptions.

## Conclusion

In summary, we provide a plausible explanation about the high frequency of the derived ADH1B*47His allele in East Asia. The distribution of the derived ADH1B*47His allele in East Asia can be well explained by the origin and expansion of the Neolithic rice culture, which is so far one of the few cases demonstrating the genetic adaptation of human populations to the dramatic change during Neolithic time. The ethanol intake increased with the origin of rice agriculture in southern China creates a selective pressure on the Neolithic populations, which is similar with the convergent adaptation of human lactase persistence in Africa and Europe along with the emergence of Neolithic cattle farming[4].

## Methods

### Samples

In this study, a total of 2,275 unrelated samples were collected from 38 populations (Table 1 and Figure 1). The Han Chinese samples were collected from individuals in 14 provincial areas whose geographic origins were assigned according to the birthplaces of their four grandparents, covering the major geographic regions in China. The other ethnic populations were sampled from south-western China and Qinghai-Tibet plateau where about 80% Chinese ethnic populations live with inhabited histories longer than 3,000 years[34]. All the samples were collected with informed consent. The protocol of this study was approved by the institutional review board of Kunming Institute of Zoology, Chinese Academy of Sciences.

### Markers and Genotyping

Initially all the samples were genotyped for the ADH1BArg47His polymorphism (rs1229984). For genotyping by PCR-RFLP, we designed primers (forward primer, 5'FAM-GATTAGTAGCAAAACCCTCAAATAC-3'; reverse primer, 5'-CTAACCATGTGGTCATCTGCG-3') to cover this region. The restriction endonuclease used is Hin61 (Fermentas Life Sciences). The two contiguous microsatellites (CATA repeats and ATTC repeats) are from the UCSC Genome Database, located on Chr: 4 100471833-1889 and Chr: 4 100423654-3678. Both the microsatellite and the SNP genotyping were carried out by using an ABI 3130 sequencer, and the data was analyzed by using Genemapper software version 3.1 (Applied Biosystems). For haplotype analysis, we used the data from HapMap (Phase2.1)[35].

### Test of Recent Selection

We used the iHS statistics[19] to detect recent positive selection. The obtained iHS value for the candidate SNP(rs1229984) and the empirical P-value for ADH1B were calculated by using Haplotter[19]. The extended haplotype homozygosity (EHH) and the relative EHH (REHH)[18] were examined by the Sweep program, using the phased haplotype data set (CHB+JPT) from the HapMap project.

### Allele Age Estimation

Allele age calculations are conducted by the standard methods published previously[24,25,36]. In brief: *t *= [1/ln(1-*c*)]ln [(*x*(t)-*y*)/(1-*y*)], where *t *= allele age (in generations), *c *= recombination rate, *x*(*t*) = frequency in generation *t*, and *y *= frequency on ancestral chromosomes. We assume the origin of the ADH1B*47His allele is on the background of the ancestral allele haplotype, and the calculation utilizes the value of c, determined from the HapMap project recombination rate database http://hapmap.ncbi.nlm.nih.gov/downloads/recombination/latest/rates/. It should be noted that the East Asian samples from the HapMap are non-family data, therefore, limiting the estimation of recombination rates in these populations. Two types of polymorphism data have been used in our analysis. The phased haplotypes (CHB) containing a 45 Kb fragment, which includes 32 SNPs and covers the ADH1BArg47His polymorphism site, is obtained from the HapMap project website http://www.hapmap.org, and the microsatellites data is collected from the randomly selected 598 individuals. For conversion of time in generations, *t*, into time in years, a generation time of 25 years is assumed.

## Authors' contributions

BS and HS designed the study; BS and YP analyzed data and wrote the paper; HS, YP, HZ, and XQ performed sample collection and genotyping; RM and CX provided part of the samples. All authors read and approved the final manuscript.

## Supplementary Material

Additional file 1 The ADH1B*47His allele frequencies and the ages of the rice relic sites in 14 regions of China. Click here for file

Additional file 2 The STR haplotypes in the 598 tested individuals. Click here for file

Additional file 3 The phased 32 SNP haplotypes of CHB. Click here for file
