# Supplementary material for: The ADH1B Arg47His polymorphism in East Asian populations and expansion of rice domestication in history
Source: BMC Evol Biol. 2010 Jan 20;10:15. doi: 10.1186/1471-2148-10-15 (PMC2823730; doi:10.1186/1471-2148-10-15)
Supplement: Additional file 1 — The ADH1B*47His allele frequencies and the ages of the rice relic sites in 14 regions of China. [file 1471-2148-10-15-S1.DOC]

**Additional file 1**

The ADH1B*47His frequencies and the ages of the rice relic sites in 14 regions of China

| **Region** | **ADH1B*47His Frequency (%)** | | **Age of rice relics** | |  |
| --- | --- | --- | --- | --- | --- |
| Zhejiang | | 98.5 | | 11,400 | |
| Hunan | | 82.5 | | 10,000 | |
| Jiangsu | | 70.8 | | 7,000 | |
| Hubei | | 70.7 | | 8,000 | |
| Guangxi | | 68.8 | | 4,700 | |
| Liaonin | | 67.9 | | 4,000 | |
| Guizhou | | 67.6 | | 3,100 | |
| Anhui | | 66.2 | | 6,000 | |
| Henan | | 64.3 | | 8,000 | |
| Shandong | | 63.3 | | 4,200 | |
| Shannxi | | 62.5 | | 5,000 | |
| Shanxi | | 61.4 | | 3,000 | |
| Gansu | | 59.8 | | 3,000 | |
| Yunnan | | 38.3 | | 3,500 | |
